# Innate Immune Cells in Melanoma: Implications for Immunotherapy

**DOI:** 10.3390/ijms25158523

**Published:** 2024-08-05

**Authors:** Marialuisa Trocchia, Annagioia Ventrici, Luca Modestino, Leonardo Cristinziano, Anne Lise Ferrara, Francesco Palestra, Stefania Loffredo, Mariaelena Capone, Gabriele Madonna, Marilena Romanelli, Paolo Antonio Ascierto, Maria Rosaria Galdiero

**Affiliations:** 1 Department of Translational Medical Sciences (DiSMeT), University of Naples Federico II, 80138 Naples, Italy; marytrocchia@gmail.com (M.T.); annagioia.v99@gmail.com (A.V.); anneliseferrara@gmail.com (A.L.F.); f.palestra97@gmail.com (F.P.); stefanialoffredo@hotmail.com (S.L.); 2 Department of Internal Medicine and Clinical Immunology, University Hospital of Naples Federico II, 80138 Naples, Italy; modestinoluca@gmail.com; 3 Center for Basic and Clinical Immunology Research (CISI), University of Naples Federico II, 80138 Naples, Italy; l.cristinziano@gmail.com; 4 Melanoma, Cancer Immunotherapy, and Development Therapeutics Unit, Istituto Nazionale Tumori IRCCS Fondazione “G. Pascale”, 80138 Naples, Italy; marilenacapone@gmail.com (M.C.); g.madonna@istitutotumori.na.it (G.M.); marilena.romanelli@istitutotumori.na.it (M.R.); p.ascierto@istitutotumori.na.it (P.A.A.)

**Keywords:** melanoma, immune checkpoint inhibitors, immunotherapy, dendritic cells, monocytes, macrophages, tumor microenvironment, neutrophils, neutrophils extracellular traps

## Abstract

The innate immune system, composed of neutrophils, basophils, eosinophils, myeloid-derived suppressor cells (MDSCs), macrophages, dendritic cells (DCs), mast cells (MCs), and innate lymphoid cells (ILCs), is the first line of defense. Growing evidence demonstrates the crucial role of innate immunity in tumor initiation and progression. Several studies support the idea that innate immunity, through the release of pro- and/or anti-inflammatory cytokines and tumor growth factors, plays a significant role in the pathogenesis, progression, and prognosis of cutaneous malignant melanoma (MM). Cutaneous melanoma is the most common skin cancer, with an incidence that rapidly increased in recent decades. Melanoma is a highly immunogenic tumor, due to its high mutational burden. The metastatic form retains a high mortality. The advent of immunotherapy revolutionized the therapeutic approach to this tumor and significantly ameliorated the patients’ clinical outcome. In this review, we will recapitulate the multiple roles of innate immune cells in melanoma and the related implications for immunotherapy.

## 1. Introduction

From a classic point of view, the innate immunity is considered the first line of protection against viruses and toxins [1]. Increasing evidence suggests a role for innate immunity in influencing the tumor microenvironment (TME) and the clinical prognosis of cancer patients [2] by promoting or inhibiting tumor initiation and progression [3]. Over the past 20 years, a number of studies revealed that innate immune cells are incredibly plastic and heterogeneous, with different phenotypes based on tumor types, stages, and therapies [4]. Indeed, much evidence supports the idea that innate immunity, through the release of pro- and/or anti-inflammatory cytokines and tumor growth factors, plays a significant role in the pathogenesis, progression, and prognosis of cutaneous MM [5]. The identification of new therapeutic targets and the development of strategies aimed at handling the interactions between immune and tumor cells could have a significant effect on immunotherapy and clinical outcomes [5].

### 1.1. Melanoma

#### 1.1.1. Epidemiology and Risk Factors

Melanoma is a malignant tumor that results from the alteration of epidermal melanocytes due to the combination of:

- Genetic factors, such as background, gender, and skin type;
- Constitutional factors, i.e., age, number, and size of pigmented nevi;
- Environmental factors, i.e., the overall lifetime exposure to solar light and sunburn frequency [6,7].

Melanoma mostly affects the skin. However, it can also develop on different mucosal surfaces, meninges, and eyes (uvea, conjunctiva, and ciliary body) [8]. Cutaneous melanoma has a growing incidence throughout the world, and numerous studies suggest that its incidence has doubled in the last 10 years [9,10]. Currently, it is expected that 99,700 new cases of melanoma in situ would be diagnosed in the skin by 2024 [11]. According to Markovic et al., males are 1.5 times more likely to get melanoma than females [12]. However, other studies suggest that the differential frequency in the two sexes should be associated with age. Indeed, up to the age of 40, females have a higher incidence compared to males; by the age of 75, the incidence of melanoma among males is about three times higher than females [13]. Regarding the diagnosis, the gold standard is represented by histopathology, with the support of immunohistochemistry [13,14,15]. The main therapeutic approach for cutaneous melanoma is surgery [16]. In recent years, targeted and immunotherapies have demonstrated effectiveness in treating advanced and high-risk resectable melanomas [17,18]. Despite these significant advances, most patients develop resistance to MAPK pathway inhibitors and immune checkpoint blockade therapy [19]. Mutations and alterations in the TME could contribute to the lack of response [20]. It is important to understand the processes by which melanomas develop resistance in order to develop personalized therapies that take into consideration each patient’s unique genetic and immunological profile.

#### 1.1.2. Molecular Pathogenesis

Normal melanocytes retain a low proliferation index but, during melanoma initiation and progression, the proliferative index rises continuously [21]. This process is accompanied by a constant increase in genetic point mutations and copy-number alterations [22]. According to cross-cancer genetic landscape investigations, cutaneous melanomas have a high frequency of ultraviolet (UV)-related mutations, such as C→T (induced by UVB) or G→T (produced by UVA), and a high mutational burden (>10 mutations per megabase) [23]. Direct mutagenic effects of UVB and UVA are the source of several relevant mutations in melanoma. Indirect effects, such as the generation of free radicals from the interaction between UVA and melanin [24], also lead to mutations and genetic aberrations [25,26].

Mutations in tumor suppressor genes and/or oncogenes frequently result in the constitutive activation of the MAPK pathway, which is also known as RAS-RAF-MEK-ERK activation cascade [27,28]. As the final result, ERK1 and ERK2 translocate to the nucleus and influence transcription factors such as MITF and c-MYC, which modify cell proliferation, apoptosis, and senescence [28,29]. Several genetic and genomic alterations in melanoma were discovered by exome and genome sequencing [30]. The most frequent mutations leading to melanoma initiation and progression include inactivating mutations of the oncosuppressor genes *TP53* and *CDKN2A*, as well as activating mutations of the oncogenes *BRAF* and *NRAS*. Up to 12% of all melanomas display multiple neurofibromin 1 (*NF1*) mutations. These mutations are more common (45%) in melanomas that are *BRAF* and *RAS* wild-type (WT) [31,32], and they are associated with a high number of inactivating mutations, including splice variants, early termination, and insertion–deletion (InDels) [32].

In 2015, the Cancer Genome Atlas Program (TCGA) Network suggested a new genomic classification for melanoma: mutant *BRAF* (31%), mutant *RAS* (18%), mutant *NF1* (21%), and triple WT (26%) [33]. The most common alterations are mutations of the serine-threonine kinase *BRAF* gene [32], leading to the constitutive activation of BRAF protein [34]. In particular, the prevalence of mutations in codon 600 of BRAF is 31% in patients with MM. The most prevalent mutations in MM are the BRAF V600E (about 80%) and BRAF V600K (5–30%), with other subtypes found at lower frequencies: V600M 4%, V600R 5%, and V600D < 5% [35]. *BRAF* mutant melanomas present as thinner tumors, display higher lymphocyte infiltration, and are detected at a younger age but generally in a more advanced stage [33]. RAS belongs to the family of proteins that bind guanosine 5′-triphosphatase (GTP). *RAS* gene mutations prevent inactivation and maintain the active state of the RAS protein [36], which leads to the phosphorylation of MEK and ERK. This process activates the MAPK pathway. *RAS* mutations typically impact the protein NRAS, most commonly in codon 61 (Q61R and Q61K), and are present in approximately 30% of melanomas [32]. In addition to the MAPK pathway, RAS is also involved in other cellular pathways, such as T-cell lymphoma invasion and metastasis 1 (TIAM1) and phosphatidylinositol-3 kinases (PI3K) [29,36]. The NF1 protein product neurofibromin stimulates the intrinsic GTPase activity of active GTP-bound RAS, leading to RAS inactivation. Mutations in the *NF1* tumor suppressor gene are the third most common group of genetic mutations. As a result of inactivating mutations, the regulatory function of NF1 is lost, leading to RAS continuous activation [37]. These mutations were found in 46.4% of BRAF and NRAS WT melanomas examined by Krauthammer et al. [31] and 14% of samples examined by TCGA [32]. *NF1* mutant tumors are more common on the head/neck in elderly patients with severe solar elastosis; they lead to bigger tumors but are diagnosed in earlier stages [33]. Remarkably, co-mutations in “*RAS*-opathy” genes (*RASA2*, *PTPN11*, *SOS1*, *RAF1*, and *SPRED1*) [38] were found in almost 60% of these melanomas [31]. Triple WT melanomas are characterized by the absence of mutations in these three genes (*BRAF*, *RAS*, and *NF1*). This subgroup consists of *KIT* or *GNAQ* mutations, which are frequently observed in mucosal and uveal melanomas. The majority of triple WT melanomas occur in males and display perineural invasion [33]. Compared to over 90% of BRAF, RAS, and NF1 subtypes, only 30% of triple WT melanomas are caused by UV [32].

About 7% to 15% of cases of melanoma occur in individuals with a family history. The most common transmission pattern of hereditary melanoma is autosomal-dominant [39]. Most cases of hereditary melanoma are due to mutations in cyclin-dependent kinase inhibitor 2A (*CDKN2A*), but other additional susceptibility genes, such as *CDK4*, *TERT*, *ACD*, *TERF2IP*, *POT1*, *MITF*, *MC1R*, and *BAP1*, have been identified recently [39]. Additionally, melanoma risk is increased in mixed cancer syndromes caused by mutations in *PTEN*, *BRCA2*, *BRCA1*, *RB1*, and *TP53*. Screening for hereditary melanoma begins with a detailed personal and family history of cancer. Historically, individual testing of susceptibility genes was performed but, in the recent decade, next-generation sequencing technologies have enabled affordable and timely testing of multiple genes [40]. In addition, a multidisciplinary team, including dermatologists, pathologists, and oncologists, is needed to identify and treat these patients. More epidemiologic, clinical, and genetic research is required to completely comprehend the mechanism of inheritance of these syndromes, the significance of these genes in patient prognosis, the impact of gene–gene interactions, and the involvement of environmental variables in gene penetrance [41].

#### 1.1.3. Immunotherapy and Melanoma

Due to its high mutational burden, immunotherapy is one of the main therapeutic approaches for MM [42]. High mutation rates raise the possibility of producing mutant proteins with potential neoantigen properties, which might lead to increased immunogenicity [43]. This likely explains the reason why immunotherapies display such exceptional efficacy in this kind of tumor [6]. The purpose of immunotherapy is to stimulate the immune system to recognize and eliminate tumor cells [44]. Over the past 30 years, immunotherapy has made progress, with a broad impact on patient survival [18]. In 1981, IL-2 administration represented the first immunotherapy attempt in melanoma [45,46]. In the mid-1990s, CTLA4 and PD1 were discovered as T-cell checkpoint receptors, which allowed the development of immune checkpoint inhibitors (ICIs) [47,48].

The first ICI approved in 2011 by the FDA was ipilimumab, an anti-CTLA4 monoclonal antibody (mAb) [49], following the results obtained in an important clinical study in 2010 [50]. Currently, ipilimumab (IPI) retains a 10-year survival rate of 20% [51]. Nivolumab (NIVO) is a humanized mAb that blocks the activation of the PD-1 receptor on the surface of T and B lymphocytes [52], with a lower rate of adverse events than adjuvant ipilimumab [53]. The 6.5-year extension of the phase III CheckMate 067 trial showed improved and durable clinical outcomes with NIVO plus IPI or NIVO alone versus IPI [54]. More recently, the 7.5-year follow-up extension showed long-lasting clinical response under NIVO + IPI combination [55]. Although ICIs have marked a turning point in the therapy of advanced melanoma, a significant fraction of patients continue to fail to respond or progress after initial response. Moreover, important immune-related adverse events can be associated with these treatments [56,57,58]. For all these reasons, it is pivotal to find efficient biomarkers that can predict patient response to treatment [59,60].

#### 1.1.4. Innate Immune System and Melanoma

The innate immune system is a first line of defense for the organism [61] through rapid recognition of pathogen-associated molecular patterns (PAMPs) and damage-associated molecular patterns (DAMPs) [62]. Increasing evidence demonstrates the crucial role of innate immunity in tumors, such as melanoma, an extremely immunogenic tumor due to its high mutational load [63,64,65]. Rapid, nonspecific responses are critical for controlling the early stages of the disease and for building a strong adaptive immune system that can provide long-term tumor-specific immunity [62]. This process gives rise to the apoptosis of cancer cells, which occurs in two phases: an early, nonspecific phase driven by dendritic cells (DCs), natural killer cells (NK), macrophages, granulocytes, and mast cells and a late phase driven by T cells producing gamma interferon (IFN-γ) [66]. Immune and tumor components communicate through exchanging signals in the tumor microenvironment (TME). Key elements of the TME include cancer-associated fibroblasts (CAFs), a population of stromal cells exhibiting phenotypic and functional heterogeneity [67]. Activated CAFs stimulate angiogenesis, invasion, metastasis, extracellular matrix (ECM) remodeling, and even chemoresistance [68] through a variety of mechanisms. A further important element in promoting tumor progression has been identified as the reciprocal effects between CAFs and the tumor immune microenvironment (TIME). The TIME is strongly correlated with the antitumor immunological status in the TME [69,70] and is primarily composed of different immune cell types within tumor islets. Through the secretion of various cytokines, growth factors, chemokines, exosomes, and other effector molecules, CAFs interact with tumor-infiltrating immune cells as well as other immune components within the TIME. This interaction results in the formation of an immunosuppressive TME that allows cancer cells to elude immune system surveillance [69,70,71].

**Figure 1:**
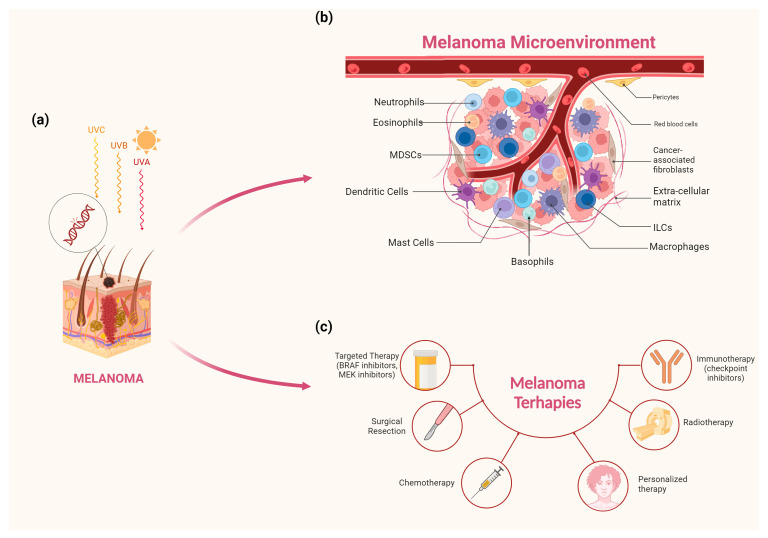
Melanoma is a malignant tumor, which develops from an alteration in epidermal melanocytes. One of the main risk factors is overall lifetime exposure to solar light and sunburn frequency. Unprotected exposure to UVA and UVB rays damages the DNA of skin cells, causing genetic defects or mutations that can lead to skin cancer and premature aging (**a**). Increasing data suggest that innate immunity has a role in affecting the tumor microenvironment (TME) and cancer patients’ clinical outcomes. Innate immune cells exhibit amazing adaptability, acquiring both pro- and anti-tumorigenic roles depending on different factors present in the TME (**b**). The primary treatment option for cutaneous melanoma is surgery. Among the treatments used against melanoma, it is also possible to find chemotherapy and radiotherapy. Melanoma cells are particularly sensitive to radiation. Until a few years ago, chemotherapy was the only weapon available in advanced disease but, today, it plays a minor role. In recent years, targeted and immunotherapies have shown promise in treating advanced melanomas. Despite these considerable gains, most people become resistant. Understanding the methods by which melanomas gain resistance is critical for developing customized medicines that take into account each patient’s unique genetic and immunological features (**c**).

A comprehensive view of melanoma features, TME, and current available therapies is summarized in Figure 1.

Understanding TIME is critical for developing cancer immunotherapies and identifying immunological regulators of cancer progression. Recent applications of single-cell RNA sequencing (scRNA-seq) in dissecting TME have yielded insights into the biology of tumor-infiltrating immune cells, including their heterogeneity, dynamics, and potential roles in disease progression and response to ICIs [72]. Immune cells respond to various immunogens in a dynamic and specialized manner due to their developmental diversity and phenotypic plasticity. Currently, flow cytometric analysis of hundreds of immune cell lineages and cell fate markers is routine, enhanced by immune status deconvolution and bulk genomic profiling of tumor tissues [73,74]. The swift advancement of scRNA-seq technologies has given rise to potent instruments for analyzing the diversity of immune cells invading tumors and their interactions with various normal and abnormal cell types [75,76,77,78,79]. Moreover, cell lineages and the dynamics of cell fate and differentiation can be followed using scRNA-seq techniques in a variety of ways. Multiple layers of information, including the epigenomic, genomic, transcriptomic, and proteomic features of individual cells and their combinations, can be collected at a resolution and cost never before possible due to the exponential growth of both technological advancements and biological throughput [75]. Studying the characteristics of immune cells, which are well known for their numerous developmental lineages, antigen specificities, phenotypic plasticity, and adaptability to different microenvironments, is perfectly suited for such high resolution [78,79]. In particular, innate immune cells are characterized by a surprising plasticity and can acquire both pro- or antitumorigenic functions depending on multiple factors present in the TME [80]. Because of the dual role of the innate immune cells in TME, our aim is to thoroughly examine the functions and the complex interplay in the melanoma microenvironment between the innate immune cells that influence the development and growth of this tumor.

**Figure 2:**
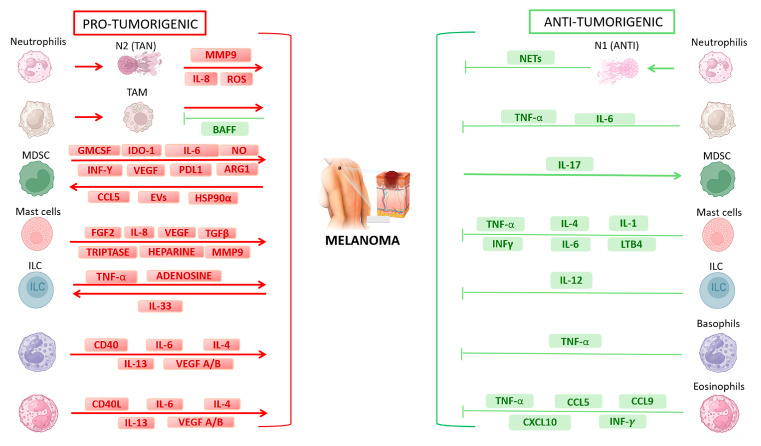
The innate immune system, composed of MDSCs, macrophages, neutrophils, DCs, MCs, basophils, eosinophils, and ILCs, is the first line of defense. Innate immune cells are characterized by a surprising plasticity and can release both pro- and antitumorigenic molecules depending on factors present in the TME. Arrows indicate protumorigenic (red ones) or antitumorigenic (green ones) effects and molecules produced by melanoma or innate immune cells within the TME.

**Figure 3:**
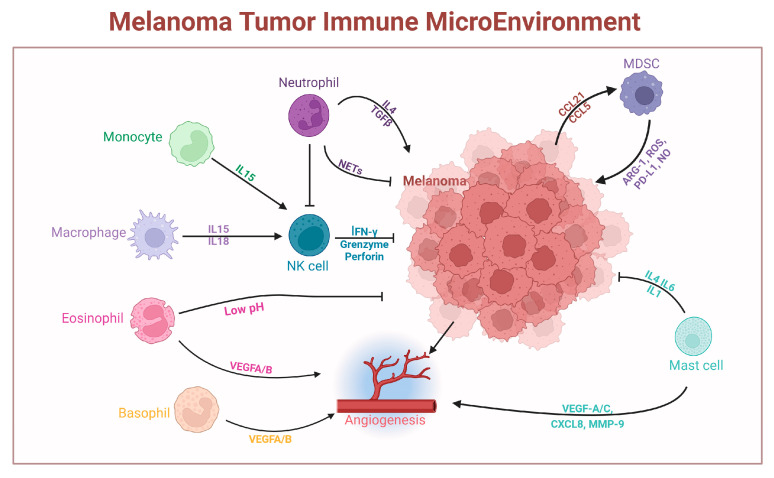
Immune cells respond to different immunogens in a dynamic and specialized manner because of their developmental diversity and phenotypic flexibility. Patrolling monocytes increase NK cell resistance to metastasis by releasing IL-15, which induces IFN-γ production. They also maintain NK cell activation by maintaining high levels of NK-cell-activating receptors and low levels of NK cell inhibitory receptors. Macrophages can activate and recruit NK cells, thus increasing their resistance to metastases. Neutrophils release neutrophils extracellular traps (NETs), which prevents the migration of tumor cells and promotes cytotoxicity towards neoplastic cells. Additionally, neutrophils promote metastasis by preventing NK cell activation basophils and eosinophils can produce various protumor signals through the release of angiogenic molecules such as vascular endothelial growth factor (VEGF) A and B. Eosinophils also display antitumorigenic activity. MDSCs are essential for the development of tumors. The interaction of CCL5 with CCR5 stimulates the growth, invasion, angiogenesis, and recruitment of immune cells into the TME. The known mediators of the immunosuppressive actions of MDSC are ARG-1, ROS, PD-L1, and NO. MCs can also produce a range of cytokines, such as TNF-α, IL-1, IL-4, IL-8, IL-6, MCP-3, and MCP-4, which can help suppress the growth of tumors by triggering apoptosis. Tumor vascularization can be promoted by MCs by the secretion of angiogenic molecules such as VEGF-A, IL-8, FGF-2, VEGF-C, MMP-2, and MMP-9.

A comprehensive portrait of the pro- versus antitumoral functions of innate immune cells in melanoma is schematically depicted in Figure 2. Figure 3 further summarizes multiple networks linking innate immune cells with each other, as well as with melanoma cells.

## 2. Innate Immune Cells in Melanoma Microenvironment

### 2.1. Monocytes and Macrophages

Because of their high plasticity, macrophages can perform a variety of functions, including immune system orchestration, tissue homeostasis, and bacterial clearance [81]. In response to various stimuli, they can differentiate into M1 classically activated macrophages and M2 alternatively activated macrophages. M2 macrophages promote tumor growth, tissue regeneration, immunological regulation, and parasite elimination [82,83,84]. Tumor-associated macrophages (TAMs) resemble a number of M2 macrophage functions and are usually classified as “M2-like” cells. TAMs can vary significantly in terms of ontogeny and functions, which are controlled by the TME [85] and their infiltration usually correlates with poor prognosis [86].

M2-like macrophages can support melanoma growth, inhibit antitumoral leukocytes, and affect the response to anti-PD1 treatment. In a murine model of melanoma graft, Wei Liu et al. developed a B16-F10 melanoma cell line that over-expressed the B-cell-activating factor (BAFF), a member of the TNF family [87]. Once implanted in recipient mice, tumors that expressed BAFF grew slower when compared to control tumors. Monocytes and TAM infiltration were reduced in BAFF-expressing tumors. In addition, monocytes from BAFF-expressing tumors displayed higher levels of proapoptotic genes and lower levels of pro-proliferative genes. These results suggest that BAFF inhibited monocytes and TAM infiltration and/or proliferation within the TME. Monocytes from BAFF-expressing melanoma showed higher levels of NF-κB signaling regulators. BAFF dampened the suppressive functions of tumor-infiltrating monocytes. BAFF levels correlated negatively with the response of patients undergoing anti-PD1 immunotherapy. For this reason, BAFF was proposed as a biomarker to forecast the response to anti-PD1 treatment [88].

M2-like macrophages can be attracted by melanoma cells. Most of our current understanding of leukocyte recruitment into cancers is based on adhesion molecules and chemokines expressed by endothelial cells (ECs). However, a mechanism known as vasculogenic mimicry (VM) enables cancer cells to form their own circulatory systems [89]. VM-competent human melanoma cells are able to induce monocyte transmigration similarly to ECs. Gene expression profiling of human melanoma patient samples indicated the presence of several leukocyte adhesion molecules and chemokines on human ECs [90,91]. Moreover, immunohistochemical staining of tissue from melanoma patients revealed a correlation between tumors with high VM content and macrophage count. Thus, regardless of an anergic EC-lined tumor vasculature, VM capillaries actively recruit monocytes into the tumor mass to enable their maturation into protumorigenic M2 macrophages [89]. Indeed, in murine models of melanoma, tumor-cell-extrinsic activation of estrogen receptor alpha (ERα) led to an increased infiltration of M2 macrophages in the TME, which, in turn, suppressed adaptive immunity and promoted tumor growth [92]. This VM mechanism can be reverted. The over-expression of IL-36α, a member of the IL-1 family [93], in melanoma cells reduced the immunosuppressive potential of the TME [94]. Indeed, IL-36α over-expression increased the recruitment of MHC II^high^ macrophages and CD8^+^ T lymphocytes and decreased the infiltration of regulatory T cells, CD4^+^ T cells, and monocytic myeloid-derived suppressor cells (M-MDSCs) in the melanoma microenvironment. These effects inhibited melanoma progression and lung metastasis. IL-36α activated the NF-κB and MAPK signaling pathways in macrophages, leading to the release of proinflammatory cytokines such as IL-1β, IL-6, TNF-α, CXCL1, CXCL2, CXCL3, CXCL5, and iNOS, thus reverting M2 macrophages into M1. Interestingly, the in vivo administration of IL-36α together with the anti-PD-L1 mAb enhanced immune cell infiltration and amplified the antitumor effect of anti-PD-L1 on melanoma growth [94].

Monocytes/macrophages also infiltrate melanoma metastatic niches and thus impact the overall survival (OS) of patients. MM is characterized by different subsets of infiltrating cells. In particular, metastatic niches can be infiltrated by CD14^+^ monocytes/macrophages with distinct transcriptional and proteomic features. The expression pattern across metastatic tumors from different metastatic niches suggests that local macrophages are reprogrammed with the arrival of metastatic melanoma cells, or that blood monocytes contribute to the macrophage pool in the metastatic sites. Indeed, some of the intratumoral CD14^+^ (iCD14^+^) transcripts were related to tissue-resident macrophages. On the other hand, monocytes present in the melanoma stroma (stromal CD14^+^— sCD14^+^) presented a transcriptional pattern different from iCD14^+^ cells. Data from the TCGA dataset indicate two patient cohorts with significantly different survival rates based on the iCD14^+^ and sCD14^+^ gene signatures. In particular, high CD14^+^LY75^+^CD2^+^ monocytes were associated to long-term survival [95].

Finally, monocytes/macrophages play an important role in supporting metastasis [91,96]. They are able to establish crosstalk with cancer cells, which attract them from the peripheral circulation. Their great plasticity allows them to be reprogrammed to an antitumor phenotype. For instance, monocytes enhance the NK-cell-mediated resistance to metastasis by inducing NK cell IFN-γ production through the release of IL-15. They also sustain NK cell activation by maintaining high levels of NK-cell-activating receptors and low levels of NK-cell inhibitory receptors [97]. Additionally, by releasing IL-15 and IL-18, macrophages can activate and attract NK cells, strengthening their resistance to metastasis [97]. For these reasons, they can be critical in the improvement of new therapeutic targets.

### 2.2. Neutrophils and Neutrophil Extracellular Traps 

Neutrophils, also known as polymorphonuclear leukocytes (PMNs), are key components of innate immunity and represent the majority of circulating leukocytes in humans [98]. They are part of the family of granulocytes, as they are characterized by a multitude of granules within the cytoplasm [99]. The production of neutrophils occurs in the bone marrow by myeloid progenitors, granulocyte monocyte progenitors (GMP), through a process called “granulopoiesis”. Neutrophils retain several strategies to attack and kill pathogens, including the release of neutrophil extracellular traps (NETs). The main process of formation of NETs, called “suicidal” NETosis, leads to the death of neutrophils and is characterized by subsequent morphological changes such as disintegration of the nuclear membrane, decondensation of the chromatin, disappearance of the plasma membrane, and, finally, the leakage of the histone core together with granular enzymes [100]. In addition to this mechanism, “vital” NETosis has been described in which neutrophils remain alive and release only parts of their nuclear or mitochondrial DNA [101].

Neutrophils operate at the interface between innate and adaptive immunity, capable of intervening as the first defense mechanism against pathogens. In addition, several studies describe the association between neutrophils and cancer [102,103,104]. The presence in the TME of cytokines or immunosuppressive molecules such as transforming growth factor beta (TGF-β) or interleukin 10 (IL-10) can favor the polarization of tumor-associated neutrophils (TANs) towards a protumor phenotype (N2), while the presence of granulocyte colony-stimulating factor (G-CSF) can promote the development of TANs with antitumor activity (N1) [105]. Interestingly, IFN-γ and tumor necrosis factor alpha (TNF-α) can convert N2 into N1 in vitro [106,107].

Neutrophils can promote tumor initiation and progression through different mechanisms. First, neutrophils recruited in the TME release proteases and produce large amounts of reactive oxygen and nitrogen species (ROS and NOS), resulting in tissue damage and chronic inflammation [108], genetic instability, and point mutations in DNA [109]. Second, neutrophils can shift their functions toward immunosuppression, promoting the inhibition of T cell immunity. Finally, neutrophils can inhibit NK cell activation and decrease NK-cell-mediated metastasis clearance, thus actively contributing to the spread of metastasis [97].

The pathogenesis of melanoma involves several events such as oncogenic mutations in progenitor cells that enhance their proliferation [110] and favor the establishment of a protumoral inflammatory environment [111]. Our group recently investigated the biological properties of the neutrophils within the context of human melanoma. The neutrophil biological properties (i.e., chemotaxis, survival, activation, cell tracking, morphology, and release of NETs) were well characterized in vitro. Indeed, cultured within conditioned media (CM) derived from the melanoma cell lines (melanoma-CM), PMNs shifted their phenotype towards an activated functional state. More in detail, under melanoma-CM stimulation, PMNs upregulated the expression of CD11b and CD66b, downregulated the expression of CD62L, modified their morphology and kinetic properties (increased their speed and straightness), and released MPO and MMP-9 in the extracellular milieu. Patients with MM also show increased circulating levels of neutrophil-derived mediators and NETs [112]. Circulating neutrophils stimulate angiogenesis and stimulate the migration of melanoma cells towards endothelial cells, promoting tumor cell invasion. NETs are capable of damaging the endothelial barrier, which promoted the metastatic spread of melanoma, favoring the entry of tumor cells into the circulation [113].

By contrast, some studies highlighted an antitumorigenic role of neutrophils and their soluble mediators in melanoma. Fiona Schedel et al. demonstrated that melanoma cells were able, through integrins, to bind NETs, which prevented the migration of tumor cells and promoted cytotoxicity towards neoplastic cells [114]. Interestingly, in a murine model of implanted melanoma, neutrophils mediated tumor eradication by melanoma-specific CD4^+^ T cell therapy in combination with OX40 co-stimulation or CTLA-4 blockade [115]. A mouse model and samples from melanoma patients treated with ICIs demonstrated abnormal neutrophil activity [115]. Transcriptomic and flow cytometry analyses revealed a distinct subset of antitumor neutrophils present in treated mice. These results revealed, in particular, an interaction between T cells and neutrophils that mediated the destruction of tumor cells [115]. Collectively, the results of these recent studies emphasize the possibility that different subsets or states of activation of neutrophils could play even opposite roles in melanoma.

With regards to the prognostic value, *BRAF* mutation detection is fundamental for therapeutic purposes, since patients can be treated with immunotherapy or targeted therapy [51]. Interestingly, in patients with stage IV melanoma, lower frequencies of PD-L1^+^ PMNs in peripheral blood were associated with improved progression-free survival (PFS) and OS compared to those with higher PD-L1^+^ PMN frequencies only in *BRAF* wild-type patient subgroup [116]. This evidence opens up new opportunities for therapeutic intervention.

### 2.3. Basophils and Eosinophils

Basophils and eosinophils are canonically considered effector cells in hypersensitivity reactions and in the defense from parasitic agents [117]. IL-3 is central for the differentiation, priming, growth, and activation of basophils [118], while IL-5 promotes the differentiation of eosinophils, in the bone marrow, starting from IL-5Rα-positive progenitors [119].

Basophils and eosinophils infiltrate human tumors. However, their mechanism of action is still poorly understood [120,121]. In fact, similarly to macrophages and neutrophils, also basophils and eosinophils display a remarkable plasticity and can possess pro- or antitumorigenic activities [122,123]. Basophils, in the TME, can produce various protumor signals through the release of different mediators such as vascular endothelial growth factor (VEGF) A and B, IL-4, LTC4, CD40L, and IL-6 [124]. However, mouse basophils can also provide antitumorigenic properties by releasing proinflammatory mediators such as TNF-α [125]. In a mouse model of melanoma, depletion of regulatory T cells was associated with increased production of IL-3, which caused basophil infiltration into the TME. Infiltrating basophils, in turn, promoted the rejection of the tumor through the release of CCL3 and CCL4. The latter chemokines, indeed, increased the recruitment of cytotoxic CD8^+^ T cells. Pharmacological depletion of basophils prevented tumor rejection [126].

Eosinophils display mainly antitumorigenic activity in melanoma. In mice implanted with B16-F10 melanoma cells, eosinophils inhibited tumor growth and lung metastasis, through the production of IFN-γ and TNF-α and skewing the polarization of M1 macrophages [127]. Moreover, eosinophils induced tumor cell killing in vitro through the production of CCL5, CCL9, and CXCL10 [128,129]. In patients with advanced melanoma, the presence of tissue eosinophils and/or peripheral blood eosinophilia is associated with response to immunotherapy and is correlated with longer survival [130]. In addition, in patients with metastatic melanoma, peripheral blood eosinophil count is associated with response to target therapy and favorable clinical outcome. Furthermore, eosinophils induced apoptosis of melanoma cells, which might be increased by further treatment with BRAF/MEK inhibitors [131].

### 2.4. Dendritic Cells

Dendritic cells serve as a link between innate and adaptive immunity, as they are the primary antigen-presenting cells (APCs) [132]. Starting from common myeloid progenitors (CMPs), present in the bone marrow, the presence of certain transcription factors, such as Nur77 [133], leads to the differentiation of monocytic DCs (moDCs). Alternatively, CMPs differentiate into the common dendritic cell progenitor (CDP), which gives rise to both conventional DCs (cDCs), subject to maturation mechanisms, and plasmacytoid DCs (pDCs) [134,135]. cDCs can differentiate into cDC1 and cDC2. cDC1s efficiently activate CD8^+^ T cells, which are critical for antitumor and antiviral immunity, while cDC2s activate Th1 and Th2 responses. pDCs are characterized by high levels of IFN-α/β upon TLR7/9 stimulation and play critical roles in viral infections [136,137,138]. DCs’ ability to deliver antigens and activate T lymphocytes makes them suitable for antitumor therapy [139]. DCs were widely used in the development of anticancer vaccines. In early clinical studies involving antitumoral DC vaccines, autologous monocytes were isolated from cancer patients and ex vivo differentiated to DCs by exposing the isolated cells to tumor-specific/associated antigens (TAA/TSA). Ex vivo matured DCs were subsequently reinfused into the patient circulation [140]. DNA vaccination (genetic immunization) is another therapeutic modality that increased antitumoral immune responses in cancer immunotherapy. In this case, the autologous DCs are first transfected ex vivo with DNA encoding the tumor antigen. Transfected cells processed the antigenic proteins and presented antigenic fragments in the context of MHC-I or MHC-II to CD8^+^ or CD4^+^ T-lymphocytes, respectively [140]. Interestingly, in vivo studies on melanoma-bearing mice revealed that the combination of tumor-selective chemotherapy with DNA vaccine targeting DCs led to total tumor eradication [141]. DCs contribute also to immunotherapy in cancer. ICI resistance was linked to a decreased tumor infiltration of effector T cells and DCs [142]. Restoration of DC function is critical to improve tumor immunogenicity. In the murine model of tg (Grm1)EPv transgenic mice, tumor progression was associated with the upregulation of checkpoint molecules and the gradual reduction in the infiltration of cDCs in the melanoma lesions. PD-L1 blockade alone did not restore antitumor immunity but activated DCs increased tumor immunogenicity. Accordingly, combining DC stimulation with antibodies against PD-1 and TIM-3 increased T cell infiltration, leading to a delay in tumor growth [143].

DCs play a fascinating role in diagnostic scenarios in melanoma. For example, reflectance confocal microscopy (RCM) has revealed that the abundance of DCs (>30%) in the dermal–epidermal junction (DEJ) is highly indicative of melanoma [144]. Peripheral blood levels of CD141^+^ DCs were lower in stage IV melanoma patients compared to healthy controls. Furthermore, anti-PD-1 therapy was ineffective in suppressing tumor growth in humanized mice, but the efficacy was increased by in vivo activating DCs with with fms-like tyrosine kinase-3 ligand (Flt3L) and a TLR3 agonist [145]. In contrast, multi-omics studies have shown that non-responder melanoma patients displayed a higher percentage of active DCs than responders [146]. Therefore, DCs could be considered biomarkers with potential predictive values in the response to immunotherapy, but their real role is still controversial.

### 2.5. Myeloid-Derived Suppressor Cells (MDSCs)

Myeloid-derived suppressor cells (MDSCs), a heterogeneous population of immature myeloid cells, play a key role in tumor progression [147,148,149]. The pattern of chemokines associated with MDSC migration to the TME depends on the tumor model and monocytic (M-MDSCs) or polymorphonuclear MDSC (G-MDSCs) subsets [150]. CCL5 can activate hypoxia-inducible factor (HIF)-1α signaling cascades, leading to increased VEGF expression [151]. HIF-1α and VEGF activate and sustain MDSCs [152]. CCL5 interaction with CCR5 promotes tumor development, invasion, angiogenesis, and immune cell recruitment to the TME [153,154]. Interestingly, both in a Ret transgenic mouse melanoma model and in melanoma patients, an accumulation of CCR5^+^ MDSCs in melanoma lesions was found. Tumor-infiltrating CCR5^+^ MDSCs displayed higher immunosuppressive activity than their CCR5^−^ counterparts [154,155]. Increased levels of various cytokines and growth factors, including IL-6, GM-CSF, VEGF, IL-1β, and IFN-γ, were described in melanoma lesions of Ret transgenic mice. These findings were correlated with the development of tumor-infiltrating MDSCs and rapid tumor progression [156]. ARG-1, ROS, PD-L1, and NO, known mediators of MDSC immunosuppressive effects [157,158,159,160,161], were expressed in CCR5^+^ MDSCs at higher levels compared with their CCR5^−^ counterparts and were linked to melanoma progression [156]. Moreover, IL-6 promoted CCR5 expression and CD8^+^ T cell immunosuppression by MDSCs [162].

Interestingly, immature myeloid cells as well as monocytes can be switched into MDSC by melanoma-derived extracellular vesicles (EVs) [163]. The term EVs is currently used to identify vesicles released by various cell types, including erythrocytes, platelets, leukocytes, and cancer cells [164]. The process of EVs secretion is particularly active in proliferating cells such as tumor cells [165]. In murine models as well as in an in vitro human setting, melanoma-derived EVs upregulated PD-L1 expression on MDSCs through a TLR4-dependent mechanism, leading to CD8^+^ T cell suppression [163,166]. Furthermore, melanoma-derived EVs contain several microRNAs (miRNAs), able to convert monocytes into MDSCs upon direct transfer. The majority of these miRNAs take part in pathways frequently linked to cancer-related immunosuppression. Transcriptional analysis allowed some miRNAs responsible for the melanoma EVs-driven transformation of monocytes into MDSCs to be identified. These miRNAs were elevated in circulating CD14^+^ monocytes, plasma, and tumor samples of melanoma patients and correlated with the myeloid cell infiltration in melanomas. Moreover, higher levels of these miRNAs were associated with shorter PFS and OS in patients receiving NIVO and IPI [167].

In addition, soluble heat shock protein (rHSP) 90α transformed human monocytes into MDSCs through TLR4 signaling. Higher levels of HSP90α in plasma of patients with melanoma correlated with augmented PD-L1 expression on circulating M-MDSC. Patients with melanoma with high levels of HSP90α displayed shorter PFS under ICIs [166]. 

MDSCs derive from GMPs, which proliferate during cancer-driven emergency myelopoiesis and express PD-1. In fact, tumor-mediated emergency myelopoiesis begins with the expression of PD-1 on myeloid progenitors. This suggests that PD-1 blockade during the early stages of melanoma may have a significant impact on antitumor immunity. Indeed, in a graft model of murine melanoma, myeloid-cell-specific PD-1 ablation boosted the functionality of T effector cells, providing antitumor protection [168].

MDSCs were associated to immunological escape and can promote metastatic spread. MDSCs were a primary source of Wnt5A, which retained direct effects on cancer cell proliferation and invasiveness in vivo. MDSC-derived Wnt5A plays also an important role in T cell suppression. Indeed, Wnt5A knockdown in myeloid cells caused a reduction in M-MDSC and Treg infiltration in the TME. Hence, targeting Wnt5A may offer an additional tool to counteract the immunosuppressive effects of MDSCs and boost immunotherapy [169].

### 2.6. Innate Lymphoid Cells

Innate lymphoid cells (ILCs) play important roles in co-ordinating the immune response, preserving homeostasis, and controlling tissue inflammation [170]. Three subsets have been identified, namely ILC1, ILC2, and ILC3, that functionally correspond to the subsets of CD4^+^ Th1, Th2, and Th17 helper T cells. IFN-γ is produced by ILC1, IL-5, and IL-13 are produced by ILC2, and IL-17 and IL-22 are produced by ILC3 [171]. ILC subsets show a great heterogeneity and plasticity in different environments [171]. Depending on the cancer type and the subset involved, ILCs can have both pro- and antitumorigenic effects [172]. Indeed, the TME might affect how ILC populations are distributed and how they function [173].

The changes in the composition of ILC subsets can be related to the TME [173]. ILC1 retain antitumor potential due to the promotion of Th1 response and to the release of IFN-γ and cytotoxic enzymes [174]. Several characteristics of NK cells and cytotoxic activity were described in the CD94^+^CD56^+^ ILC1-like subset [175,176]. By contrast, in the melanoma TME, TGF-β converted NK cells into noncytotoxic ILC1-like cells, which had an immune-suppressive phenotype that aided in metastasis through the release of TNF-α [177,178]. In addition, DCs express proinflammatory cytokines, causing ILC1-like cells to release PDGF and GMCSF, which stimulated tumor angiogenesis [179].

Several cancer types display an increased ATP catabolism, leading to high levels of adenosine [180,181]. CD73 is an extensively expressed ectoenzyme capable of inducing the dephosphorylation of ATP to adenosine, which has strong immunosuppressive properties and has been connected to enhanced tumor development and metastasis. The presence of CD73 on ILCs supports the idea that these cells play a role in the production of the immunosuppressive metabolite adenosine [172,173]. Since human ILCs express the high-affinity adenosine A2A receptor, the nucleoside might also directly inhibit ILCs [182]. This pathway could be influenced by IL-33 [183]. IL-33 is able to induce the expression of CD73 in ILC2. Thus, IL-33 could play a role in balancing pro- and antitumor immune components within the TME through this mechanism [184]. Indeed, in melanoma, IL-33 stimulates the growth of Lin^−^ CD25^+^ ILC2s, which produce IL-5/IL-13. These cytokines are associated with a tumor-promoting microenvironment [184]. Moreover, tumor-infiltrating ILC2s (TILC2s) express PD-1 [185] and are still able to proliferate even under anti-PD-1 inhibition [186]. The anti-PD-1 mAb NIVO caused a reduction in the total number of ILCs in the peripheral blood of melanoma patients, together with the increase in the mature CD117^−^ILC2 cell subset. A higher PFS was correlated with lower peripheral frequencies of mature CD117^−^ILC2 in melanoma patients under NIVO [187]. By contrast, ILC2 can also have an antitumorigenic role. In murine models, ILC2s promoted peripheral blood eosinophilia, which sustained immune surveillance against melanoma [185,188]. In addition, by producing IL-5, ILC2 causes the recruitment of eosinophils to the lung metastasis site. By lowering glucose levels and increasing the acidity of the metastatic microenvironment, eosinophils control metastasis growth [189]. Eosinophils surround the metastasis once they enter the lungs and prevent it from spreading [188]. Interestingly, Lim et al. identified a new ILC subset implicated in melanoma: Tbet^+^NK1.1^−^ ILCs [190]. Tumor-derived lactate increased the expression of PD-1 on Tbet^+^NK1.1^−^ ILCs in the TME, which led to an increase in fatty acid metabolism and a reduction in the mammalian target of rapamycin (mTOR) signaling [191]. PD-1-deficient Tbet^+^NK1.1^−^ ILCs express higher levels of IFN-γ and granzyme B. Furthermore, in an experimental mouse model of melanoma, PD-1-deficient Tbet^+^NK1.1^−^ ILCs impaired tumor growth [190].

**Figure 4:**
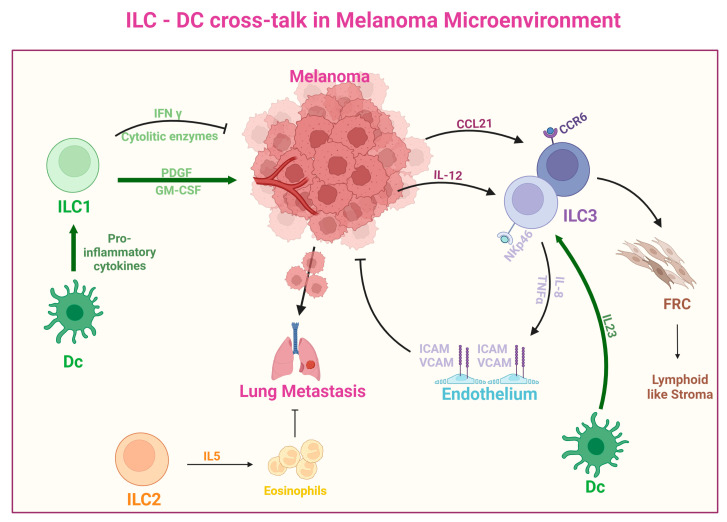
ILC and DCs cross-talk in melanoma microenvironment. Proinflammatory cytokines expressed by DCs trigger the production of PDGF and GM-CSF by ILC1 cells, which, in turn, promotes tumor angiogenesis. ILC2 induces the recruitment of eosinophils to the lung metastatic niche, through the production of IL-5. Melanoma secretes IL-12, which causes endothelial cells to upregulate ICAM and VCAM, thus attracting NKp46+ ILC3 to the tumor bed and activating the vasculature. By promoting leukocyte infiltration through endothelial activation, NKp46+ ILC3 act against melanoma. Melanoma secreting CCL21 attracts CCR6^+^ (CD4^+^) ILC3, which interact with fibroblastic reticular cells (FRC) to produce lymphoid-like stroma, generating a tolerogenic tumor environment. ILC3 are activated by DC-derived IL-23, through the expression of RORγt. Thus, when DC-derived IL-23 is produced in the melanoma microenvironment, ILC3s are activated and contribute to the protection against melanoma.

In the graft model of melanoma, IL-12 promoted the expression of Tbet in ILC3, leading to the development of “ex-ILC-3s”, which have a phenotypic similarity to ILC-1 and displayed antitumor functions [190]. Moreover, DC-derived IL-23 activate ILC3 [192] in the melanoma microenvironment, inhibiting melanoma growth and progression [193]. In addition, melanoma-derived IL-12 attracts NKp46+ ILC3 to the tumor bed and activates the vasculature by prompting endothelial cells to express ICAM and VCAM. Endothelial activation, in turn, increases leukocyte chemotaxis and inhibits melanoma growth [189,194]. By contrast, melanoma-derived CCL21 attracts CCR6^+^ (CD4^+^) ILC3, which interacts with fibroblastic reticular cells (FRC) to form lymphoid-like stroma, creating a tolerogenic tumor environment [189]. A complete description of the cross-talk between DCs, ILCs, and melanoma is shown in Figure 4.

### 2.7. Mast Cells

Several tumors are associated with the accumulation of peritumoral and/or intratumoral mast cells [195,196,197], suggesting that these cells can help to promote and/or limit tumorigenesis. Similarly to macrophages and neutrophils, it has been suggested that two major subsets of mast cells, namely MC1 (antitumorigenic) and MC2 (protumorigenic), could play distinct or even opposite roles in tumorigenesis [198]. Indeed, mast cells are characterized by functional plasticity [199] and they can be associated with both favorable and unfavorable prognosis [198,200].

On the one hand, MCs are also a source of a variety of cytokines, including IL-1, IL-4, IL-8, IL-6, MCP-3, MCP-4, and TNF-α, which contribute to the inhibition of tumor development [201].

In melanoma, MCs exert an antimetastatic role by inhibiting a nuclear protein, the high mobility group AT-hook1 (HMGA1), that binds to the AT-rich DNA strands and regulates the transcriptional activity of several genes involved in tumor cell proliferation [202]. High HMGA1 expression in metastatic tissue is related with reduced OS, whereas the reduced expression of HMGA1 limited the metastatic behavior of melanoma cells [203].

On the one hand, in the TME, MCs can release angiogenic molecules such as VEGF-A, CXCL-8, FGF-2, VEGF-C, MMP-2, and MMP-9 [198], thus promoting tumor vascularization and invasiveness [204]. Tumor-infiltrating MCs are also implicated in therapy resistance and tumor progression [205]. The presence of tumor-infiltrating MCs is associated with HLA class I down-modulation on tumor cells, the absence of CD8^+^ T cells, and immunological escape after anti-PD-1 therapy. Following anti-PD-1 therapy, there is an increase in chemokine production, leading to increased infiltration of MCs into the tumor. The combination of sunitinib or imatinib, both receptor tyrosine kinase inhibitors, and anti-PD-1 led to complete tumor regression, indicating that mast cell depletion is beneficial for responses to ICI therapy [206]. Cutaneous MCs contain and release both chymase and tryptase in their secretory granules [207]. MCs chymase is a potent serine proteinase with negative effects on keratinocytes adhesion and migration [208]. These properties suggest that it may also be involved in the migration, adhesion, and spreading of melanoma cells [209]. Indeed, recombinant human chymase promoted detachment of adherent melanoma cells and tumor spread [207].

Melanoma patients treated with anti-CTLA-4 and anti-PD-1 mAbs who developed immune-related enterocolitis had a better clinical response and OS compared to subjects who developed other adverse events (AEs) [210]. Increased serum levels of LPS in these patients were associated with increased mast cell infiltration of melanoma. The authors suggest that LPS-mediated TLR4 activation of mast cells induced the release of CXCL10, a chemokine that attracts effector T cells to tumor. These results were extended in animal models of B16-OVA melanoma [210].

Given the MCs plasticity and the variety of mediators they release, which have wide-ranging and even contradictory effects, disagreement about the functional significance of MCs in many cancer types, including melanoma, is not surprising.

## 3. Conclusions

The OS of patients with advanced melanoma has improved significantly in recent years, despite an increasing incidence that is probably attributable to improvements in early diagnosis. Indeed, the introduction of immunotherapy has represented a revolution in the therapeutic strategies of melanoma and significantly prolonged patient survival. Finding patient subgroups most likely to benefit from immunotherapy is of great interest, though, as some patients continue to fail or worsen despite initial therapy.

Tumor immune evasion through acquired or primary resistance to immunotherapies is a significant factor that lowers response. Novel therapeutic techniques are required to improve the OS rate of patients with melanoma by overcoming primary resistance and reducing the risk of acquired resistance. Identification and comprehension of the pathways that lead to tumor immune evasion during immunotherapy have come a long way in recent years. Several mechanisms were identified essentially related to adaptive immunity. More recently, an active role of innate immune cells has been described in melanoma. In this review we recapitulated all the mechanisms related to the functions of innate immune cells in melanoma, with particular reference to immunotherapy. This evidence highlights the potential roles of innate immune cells in multiple scenarios. On one hand, they can be helpful as predictive biomarkers of ICIs response. On the other hand, they can actively take part in melanoma progression and can be involved in the mechanisms related to ICIs response or resistance. Compared to the adaptive counterpart, the real knowledge of the roles of innate immunity in melanoma immunotherapy is still in its infancy.

In an even more patient-tailored point of view, further efforts are needed to deeply understand the detailed definition of the innate immune cell functions in advanced melanoma. The acquisition of this new evidence could pave the way to the identification of predictive biomarkers for treatment response, as well as to the development of new immunotherapeutic tools able to further improve melanoma patient clinical outcome.
